# Typical endobronchial carcinoid tumor in a 15-year-old female: A case report

**DOI:** 10.1016/j.radcr.2025.03.071

**Published:** 2025-05-02

**Authors:** Valeria Del Castillo, Laura Manuela Olarte Bermúdez, Arash Stephen Jalisi, Angela Moreno Sarmiento, Bibiana Pinzón

**Affiliations:** a Department of Diagnostic Imaging, Fundación Santa Fe de Bogotá, Bogotá, Colombia; b Diagnostic Radiology Residency Program, Universidad El Bosque, Bogotá, Colombia; c School of Medicine Program, Universidad de Los Andes, Bogotá, Colombia

**Keywords:** Neuroendocrine tumor, Carcinoid tumor, Pediatric oncology

## Abstract

Neuroendocrine neoplasms (NENs) are rare pathological entities arising from neuroendocrine cells, predominantly found in the lungs, pancreas, and gastrointestinal tract. Although their prevalence in children and adolescents is low (approximately 0.5 cases per million population), pulmonary carcinoid tumors represent the most common primary lung neoplasms in the pediatric population in the United States. We present the case of a 15-year-old female with hemoptysis, right hypochondrial pain, and chronic cough. Chest radiography revealed a well-defined hilar mass, prompting further evaluation with computed tomography (CT), demonstrating a hyperattenuating lesion in the right intermediate bronchus, causing partial bronchial obstruction. Diagnosis of a typical carcinoid tumor was confirmed following bilobectomy, necessitated by intermediate bronchus involvement.

## Introduction

Carcinoid tumors are rare malignant neuroendocrine neoplasms originating from enterochromaffin cells, accounting for less than 1 of all lung tumors, with a reported bronchopulmonary incidence ranging from 0.22 to 1.57 per 100,000 individuals [1]. In the pediatric population, pulmonary carcinoid tumors are the most common primary lung neoplasms, albeit with an exceptionally low prevalence of approximately 0.5 cases per million inhabitants [2]. These tumors are histologically classified as typical or atypical carcinoids, based on mitotic count and the presence or absence of necrosis. Typical carcinoids are characterized by indolent behavior and low metastatic potential, whereas atypical carcinoids exhibit more aggressive features and a higher likelihood of metastasis [3].

Although pulmonary carcinoid tumors are more frequently diagnosed in young adults, their occurrence in pediatric patients poses a significant diagnostic challenge due to their nonspecific clinical presentation, which often mimics other respiratory conditions such as asthma, pneumonia, or foreign body aspiration. This report highlights the radiological findings, clinical presentation, and multidisciplinary approach in the diagnosis and management of a pediatric patient with a typical endobronchial carcinoid tumor. By emphasizing key imaging features and management strategies, this case contributes to the limited body of literature on pulmonary carcinoid tumors in children, aiming to increase awareness and improve diagnostic accuracy for this rare entity in the pediatric population.

## Case report

A 15-year-old female presented to the emergency department with a history of chronic cough, hemoptysis, and right hypochondrium pain. The patient had a history of asthma diagnosed at the age of 2 and a paternal family history of cancer. This was the third episode of similar symptoms within the past year. On physiscal exam, neck without adenopathies or masses, mobile, pulmonary fields without wheezing, with decreased air entry at the right lung base and neurological examination normal.

**Fig. 1:**
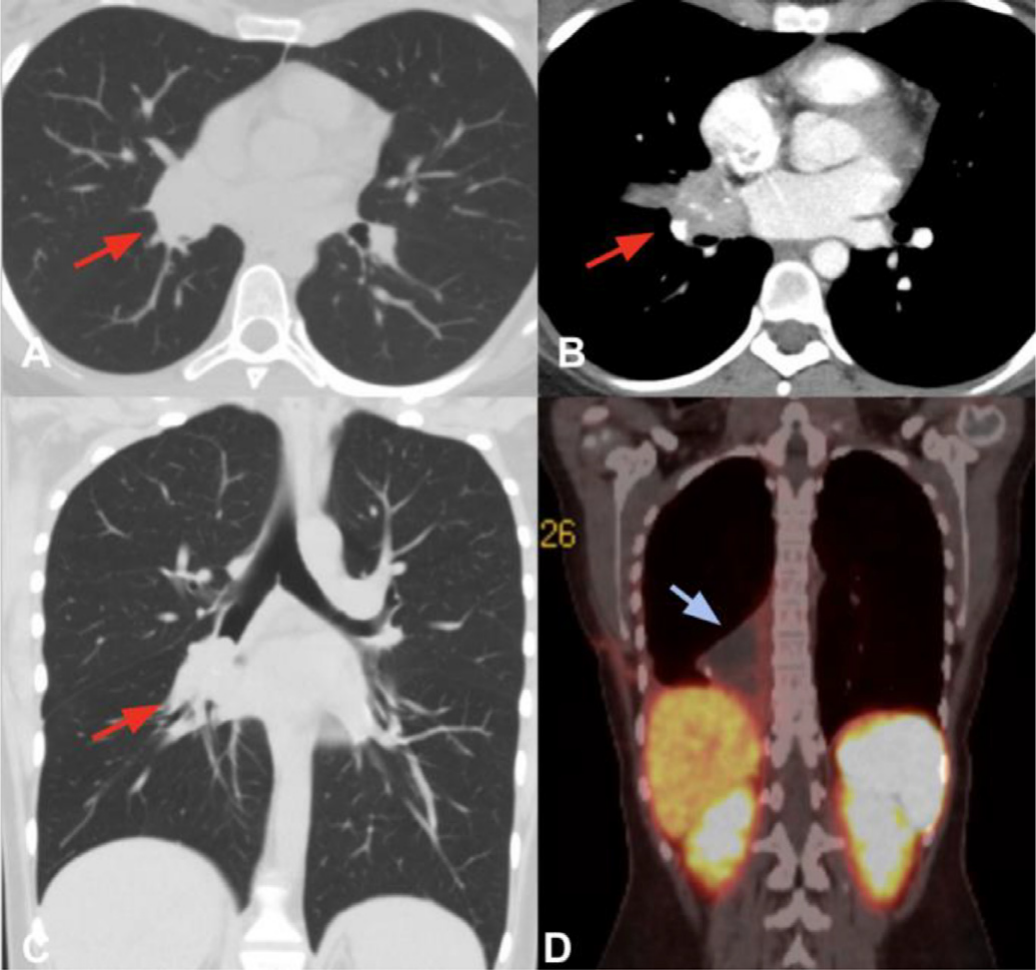
Computed tomography of thorax in axial (A) and coronal (C) sections in lung window without contrast shows a parahilar mass with regular borders and in an axial section with contrast in arterial phase (B) shows calcifications in its interior involving the right bronchus intermedius (red arrow). Postsurgical Ga68-Dotanoc PET/CT (D) without evidence of somatostatin receptor overexposure lesions shows nodule (white arrow) with moderate somatostatin receptor expression (Krenning 2), located in the retroareolar region of the right breast of 28 mm (SUVmax 5.5).

Initial laboratory investigations revealed results within normal limits, with no evidence of infection or systemic inflammation. A contrast-enhanced computed tomography (CT) scan demonstrated a 2.7 cm right parahilar nodular lesion with soft tissue density, internal calcifications, and prominent postcontrast enhancement. The lesion caused complete obstruction of the right main bronchus and bronchus intermedius, resulting in postobstructive atelectasis and mucoid impaction (Fig. 1).

The pediatric pulmonology team re-evaluated the patient, who reported a weight loss of 5 kg over the past 6 months. Given the previous findings, a differential diagnosis of bronchial adenoma versus carcinoid tumor was considered, prompting additional studies, including laboratory tests to rule out carcinoid syndrome and tumor lysis syndrome, pulmonary function tests (PFTs) with pulmonary scintigraphy, and a scheduled fibrobronchoscopy with bronchial lavage. During the fibrobronchoscopy, a rounded mass was identified within the right bronchial tree, specifically in the middle bronchus, completely obstructing the passage of the bronchoscope. Additionally, purulent material was observed draining from the bronchus.

A pulmonary perfusion scan (MAA-Tc99m) was performed, revealing normal tracer uptake in the left lung, severe uptake reduction in the right lower lobe, and moderate reduction in the right middle lobe. Pulmonary function was assessed, showing the following distribution: in the right lung—upper third 17.60, middle third 18.36, lower third 4.75, with a total of 40.7; and in the left lung—upper third 17.66, middle third 27.34, lower third 14.29, with a total of 59.29. Spirometry was performed, revealing pulmonary obstruction and restriction that were not reversible with a bronchodilator. Pulmonary volumes showed air trapping, also not reversible with a bronchodilator. Given these findings, a surgical board meeting was held, during which the pediatric surgery and thoracic surgery teams indicated surgery due to the high risk of occupation or complete compression of the right main bronchus.

**Fig. 2:**
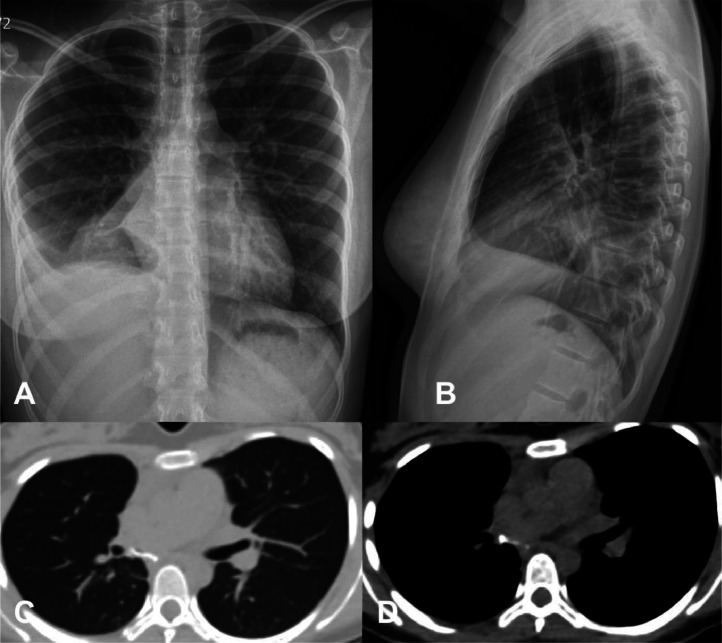
Posteroanterior (PA) and lateral thoracic radiographs (A-B) demonstrate postsurgical changes following a right lobectomy, with obliteration of the right costophrenic angle due to a larger volume of effusion on the right side and partial atelectasis of the middle lobe. Axial chest CT images in lung windows without contrast (C-D) confirm postsurgical changes, including volume loss and a rightward mediastinal shift. A triangular-shaped paracardiac opacity is consistent with partial middle lobe atelectasis. Additionally, obliteration of the right costophrenic angle is observed, likely secondary to residual pleural effusion.

Consequently, the patient underwent an emergent thoracotomy with pulmonary bilobectomy of the right middle and lower lobes and complete resection of the malignant tumor. The procedure was successfully performed without complications. A postoperative chest X-ray revealed right lung volume loss due to prior lobectomy, with a suture visible over the pulmonary base. A triangular-shaped right paracardiac opacity was noted, consistent with partial middle lobe atelectasis. Additionally, obliteration of the right lateral and posterior costophrenic angles was observed, though reduced compared to the previous study, likely due to resolving pleural effusion (Fig. 2).

**Fig. 3:**
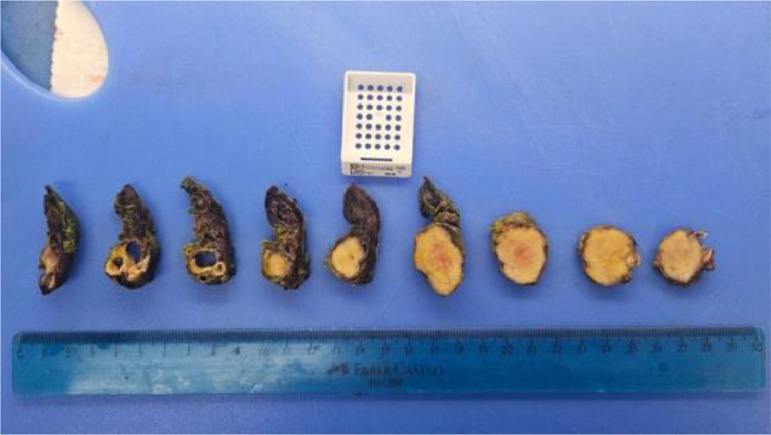
Gross specimen of the resected typical pulmonary carcinoid tumor, showing a whitish, elastic mass measuring 2.7 × 2.7 × 2 cm. On sectioning, the tumor is seen extending from the bronchial margin with significant endobronchial involvement, resulting in complete obstruction of the bronchial lumen.

In the pathology laboratory, the specimen was labeled as “right pulmonary hilum tumor” and consisted of a bilobectomy product weighing 101 grams and measuring 13.2 × 8.5 × 4.4 cm. At the level of the bronchial section margin, a whitish, elastic mass measuring 2.7 × 2.7 × 2.2 cm was identified, macroscopically in contact with the pleura. On sectioning, the mass extended from the bronchial margin with endobronchial involvement, completely occupying the bronchial lumen. No hemorrhage or necrosis was observed macroscopically. Adjacent to the tumor, dilated bronchial structures were observed in an area measuring 2.4 × 2.0 × 1.8 cm, with the largest bronchial diameter measuring 1.5 cm (Fig. 3). The remaining pulmonary parenchyma showed a loss of crepitance but no additional lesions. On microscopic examination, the sections revealed a tumor composed of nests and trabeculae arranged in an organoid pattern. The tumor cells displayed ovoid nuclei with characteristic salt-and-pepper chromatin and scarce to moderate amounts of cytoplasm. No foci of necrosis were observed. A single mitotic figure was identified in 2 mm² (Fig. 3).

Immunohistochemical studies demonstrated tumor cell reactivity for chromogranin and synaptophysin. Additional findings included a dot-like pattern of reactivity for CAM 5.2 and negative for TTF1. No lymphovascular invasion or visceral pleural involvement was identified. However, the bronchial section margin showed tumor involvement. Based on the morphological and immunophenotypic findings, a diagnosis of a typical carcinoid tumor was confirmed. Following the surgical procedure and a satisfactory clinical recovery, the patient had an adequate clinical evolution, with no respiratory difficulty, no need for oxygen support, and no pain. Therefore, discharge was indicated, with continued management by pediatric oncology and neumology.

## Discussion

Typical endobronchial carcinoid tumors (ECTs) are rare neuroendocrine neoplasms classified as typical or atypical based on histopathological features, according to the World Health Organization and the International Association for the Study of Lung Cancer (WHO/IASLC) criteria, which include mitotic count and the presence or absence of necrosis [3]. The prevalence of neuroendocrine neoplasms (NENs) in the pediatric population is estimated at 0.5 cases per million inhabitants, with pulmonary carcinoid tumors being the most common primary pulmonary neoplasms in children [4]. Although rare, these tumors warrant attention due to the potential for delayed diagnosis and their significant impact on patient outcomes.

The clinical presentation of ECTs often includes recurrent pneumonia, hemoptysis, localized wheezing, and persistent cough, typically attributable to bronchial obstruction. Imaging plays a pivotal role in their diagnosis [5]. On chest X-rays and CT scans, central bronchial carcinoids commonly present as hilar or perihilar masses, rounded or ovoid in shape, with a close anatomical relationship to the bronchus, frequently causing obstructive phenomena. In contrast, peripheral carcinoids present as solitary nodules without evidence of airway obstruction. Additional imaging findings may include atelectasis, air trapping, or mucoid impaction [2].

The initial diagnostic modality of choice is CT, as it provides detailed information regarding the tumor's local extent and lymph node involvement, enabling differentiation between typical and atypical bronchial carcinoids [7]. High-resolution CT performed during the expiratory phase can further enhance diagnostic accuracy by revealing findings such as mosaic attenuation or air trapping, both indicative of airway obstruction. Bronchoscopy, often performed in conjunction with imaging, plays a crucial role in obtaining tissue samples for histological confirmation, solidifying the diagnosis, and guiding subsequent management [6]. The imaging features of ECTs overlap with other airway lesions, making differentiation crucial:

• Mucoepidermoid carcinoma (MEC): Another primary airway tumor in children, often indistinguishable from ECTs on CT, but it can demonstrate heterogeneous enhancement and nodal involvement [6].
• Foreign body aspiration: Can mimic airway obstruction but typically has a history of sudden-onset symptoms and is diagnosed via bronchoscopy.
• Granulomatous infections: Conditions such as tuberculosis or histoplasmosis may cause localized airway narrowing and postobstructive changes [2].

Positron emission tomography (PET) using F-18 fluorodeoxyglucose (FDG) is commonly utilized in the evaluation of thoracic malignancies. However, its utility is limited for low-grade tumors, such as typical carcinoids, due to their low metabolic activity and minimal FDG uptake. In this case, FDG-PET was not performed, as it would not have altered the management plan, which involved urgent curative surgical resection.

Follow-up imaging is essential for monitoring recurrence and long-term outcomes in patients with ECTs. While CT remains the primary modality for surveillance, PET imaging using Gallium-68 DOTATATE PET/CT has emerged as a superior alternative to FDG-PET for detecting residual disease and distant metastases, given its higher sensitivity for neuroendocrine tumors [7]. Current guidelines recommend postoperative imaging at regular intervals, typically with a chest CT every 6-12 months for the first 2 years, followed by annual surveillance for up to 10 years. In cases with atypical features or suspected recurrence, DOTATATE PET/CT should be considered to guide further management [8].

The treatment of choice for ECTs is surgical resection, as seen in this case, where the patient required complete tumor excision. The prognosis of ECTs is closely linked to the pathological grade and stage of the tumor. Five- and ten-year survival rates for typical and atypical carcinoids are 90, 80, 70, and 50, respectively [9]. However, less invasive treatment approaches, such as endobronchial therapies, may be considered due to their low-grade malignancy potential.

This case highlights the importance of early imaging recognition and multidisciplinary collaboration in the diagnosis and management of pediatric airway tumors. Radiologists play a key role in identifying indirect signs of bronchial obstruction, prompting further workup, and guiding clinicians toward timely intervention.

## Conclusions

This case highlights the critical role of radiologic imaging, particularly CT and HRCT, in diagnosing typical endobronchial carcinoid tumors (ECTs) in pediatric patients. Key imaging findings, such as bronchial obstruction and postobstructive complications, are crucial for timely surgical intervention, the gold standard for treatment. Given the rarity of ECTs in children, maintaining a high index of suspicion in cases of recurrent or unexplained respiratory symptoms is essential.

Radiologists should be vigilant for indirect signs of endobronchial lesions, such as persistent atelectasis, recurrent pneumonia, or air trapping, prompting further investigation with contrast-enhanced CT or bronchoscopy. Early recognition and accurate imaging interpretation significantly impact patient outcomes, facilitating prompt surgical resection and reducing long-term complications. This case underscores the indispensable role of radiology in the multidisciplinary management of pediatric airway tumors, reinforcing the need for a proactive diagnostic approach to ensure optimal patient care.

## Patient consent

The author(s) should confirm that written informed consent has been obtained from the involved patient(s) or if appropriate from the parent, guardian, power of attorney of the involved patient(s); and, they have given approval for this information to be published in this case report (series).

Complete written informed consent was obtained from the patient for the publication of this study and accompanying images.
